# Crop-saving with AI: latest trends in deep learning techniques for plant pathology

**DOI:** 10.3389/fpls.2023.1224709

**Published:** 2023-08-01

**Authors:** Zafar Salman, Abdullah Muhammad, Md Jalil Piran, Dongil Han

**Affiliations:** Department of Computer Science and Engineering, Sejong University, Seoul, Republic of Korea

**Keywords:** deep learning, disease detection, computer vision, machine learning, plant disease, vision transformers

## Abstract

Plant diseases pose a major threat to agricultural production and the food supply chain, as they expose plants to potentially disruptive pathogens that can affect the lives of those who are associated with it. Deep learning has been applied in a range of fields such as object detection, autonomous vehicles, fraud detection etc. Several researchers have tried to implement deep learning techniques in precision agriculture. However, there are pros and cons to the approaches they have opted for disease detection and identification. In this survey, we have made an attempt to capture the significant advancements in machine-learning based disease detection. We have discussed prevalent datasets and techniques that have been employed as well as highlighted emerging approaches being used for plant disease detection. By exploring these advancements, we aim to present a comprehensive overview of the prominent approaches in precision agriculture, along with their associated challenges and potential improvements. This paper delves into the challenges associated with the implementation and briefly discusses the future trends. Overall, this paper presents a bird’s eye view of plant disease datasets, deep learning techniques, their accuracies and the challenges associated with them. Our insights will serve as a valuable resource for researchers and practitioners in the field. We hope that this survey will inform and inspire future research efforts, ultimately leading to improved precision agriculture practices and enhanced crop health management.

## 1 Introduction

More than 58% of the world’s population works in agricultural industries. In India, about 70% of small households depend upon agriculture (Government of India, 2020). A widespread disease in plants poses a potential threat to not only the livelihood of thefarmers but also to the ones consuming the crops. To protect the crop yield, early disease diagnostics are necessary. According to the Food and Agriculture Organization of the United Nations, plant diseases have increased in recent years due to climate change (Oerke and Dehne, 2004). This poses a serious risk to the livelihood of the population associated with farming and the consumers. This risk can be minimized by early detection of diseases in plants which is conventionally done by the visual inspection of a Plant. A diagnosis of a particular disease depends upon the knowledge and expertise of the inspector (Shirahatti et al., 2018). This becomes a challenge for small-scale farmers who do not have access to an expert as these methods are expensive and time consuming. In the past, scientists typically applied large-scale genetic screening and genomic approaches to identify genes and proteins of interest. These studies provided knowledge on plant behavior in response to an infection. Researchers gathered a large amount of data on the behavior and visuals of an infected plant and used digital image processing techniques to identify behavioral patterns of plants in response to a disease. Many researchers have proposed automatic recognition of disease in plants to overcome problems associated with unavailability of resources for disease detection (Oerke, 2006). With recent advancements in technology, researchers have made use of Machine Learning and Deep Learning to not only identify genes/proteins involved in plant-pathogen interactions (Danilevicz et al., 2022; Ilyas et al., 2022), but also to classify plant diseases from images of infected leaves, stems and roots. Machine Learning is the use and development of computer systems that are able to learn and adapt without following explicit instructions, by using algorithms and statistical models to analyze and draw inferences from patterns in data. Some traditional machine learning techniques that have been used in the past for disease detection in plants include Support Vector Machines (Rumpf et al., 2010), Naïve Bayes (Sperschneider et al., 2016), random forest (Ramesh et al., 2018) and K-nearest neighbors (Resti et al., 2022). However, these conventional Machine Learning approaches performed well under limited circumstances only (Nigam and Jain, 2019).

With the technological advancements in computational power, Deep learning, a subset of machine learning, has gained popularity among researchers for disease identification and classification. Deep Learning is a branch of machine learning composed of a number of algorithms that try to model high-level data abstractions using a deep graph with several processing layers containing linear and non-linear transformations. Deep Learning techniques, including Convolutional Neural Networks (CNN) for image classification, object detection and semantic segmentation have emerged as the most promising approaches given their ability to learn reliable and discriminatory visual characteristics. These techniques have shown success in various applications of computer vision such as instance segmentation and detection. However, deep learning is data-hungry and relies on large datasets consisting of hundreds and thousands of images.

### 1.1 Related work

Scientists are studying to address the problems related to plant disease and these studies indicate a rising need of an affable approach of identifying a plant disease through the use of a stand-alone device which would eliminate the need for an expert’s analysis (Mohanty et al., 2016). To accomplish this task, a large image dataset is required for Deep Learning models to train and classify healthy and diseased plants (Katal et al., 2022). To explore plant pathology studies and the application of deep learning techniques, we conducted a keyword analysis using terms such as “Plant,” “Disease,” “Defects,” “Computer vision,” “Machine Learning,” “Deep Learning,” and “Image Processing.” We performed searches on Scopus and extracted relevant research papers, which were used to generate a network visualization map for insights.

**Figure 1:**
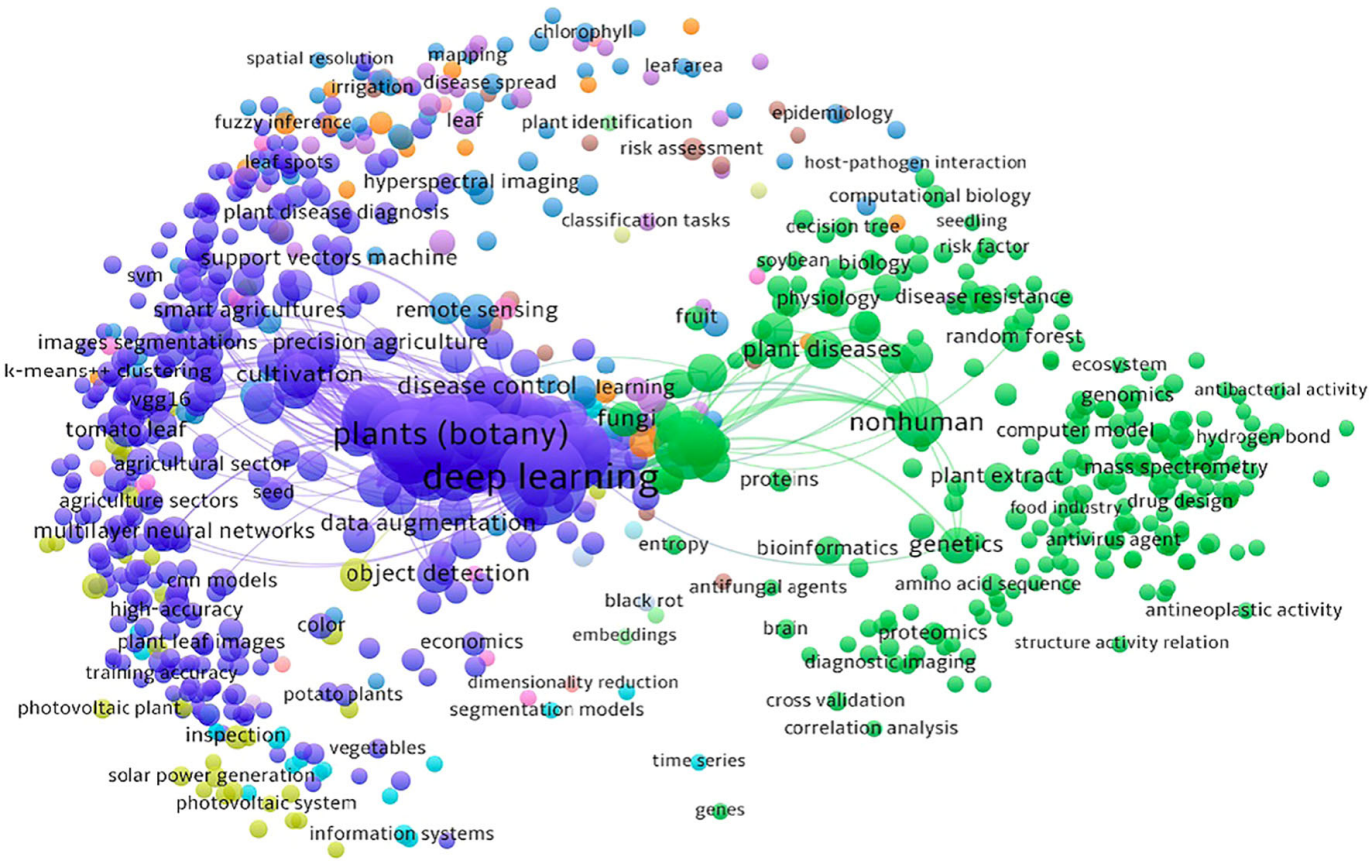
A co-word visualization illustrating the research landscape and interplay between computer vision, deep learning technologies and agricultural challenges through an analysis of keywords in research papers.

The network visualization map shown in **Figure 1** was generated by VOSViewer (Van Eck and Waltman, 2011) bibliometric software illustrates a co-word visualization. Co-word visualization is a technique used to represent the relationships between the keywords in a dataset where each circle represents a keyword and each line represents the relationship between the keywords. The size of a point is directly proportional to the presence of that keyword in the analyzed data indicating its occurrence frequency.

As seen in **Figure 1**, there are two main clusters: one related to deep learning and the other related to plants (botany). These clusters suggest that a significant amount of work has been done in the field of plant pathology using deep learning techniques.

The sub-clusters within the deep learning cluster contain keywords that are closely related to various topics such as cross-validation, neural networks, SVM, VGG16, and more. These keywords indicate specific areas or techniques within the field of deep learning that have been explored in the context of plant pathology. The second main cluster, focused on plants (botany) also consists of several sub-clusters that branch out of it. These sub-clusters represent keywords related to different aspects of plant pathology. For instance, there are keywords related to antibacterial activity, plant disease diagnosis, black rot, tomato leaf and others. By visually analyzing the co-word visualization, one can gain an intuitive understanding of the main themes or topics covered in the analyzed data. These clusters provide insights and highlight the relationships and importance of various concepts within the field of plant pathology and deep learning.

In this survey, we reviewed the most recent and most cited survey papers that are published since 2019. **Table 1** shows notable contributions of 11 most cited survey papers as per Scopus index which considered different aspects of disease diagnostics in Plants while **Table 2** represents the most recent surveys ranked on Scopus index. In 2019, a comprehensive survey on using deep learning for image-based plant disease detection was conducted by S.P. Mohanty (Kaur et al., 2019) in which various studies were performed for the automation of identification and classification of plant disease using machine learning and image processing techniques. Their survey demonstrates the effectiveness of convolutional neural networks (CNNs) in accurately identifying and classifying plant diseases. The key findings of the study include the superior performance of deep learning models compared to traditional machine learning approaches, the importance of dataset quality and diversity for training robust models, and the potential of transfer learning to overcome limited data challenges in plant disease detection. Kaur et al. did a similar survey on plant disease identification and classification through leaf images (Li et al., 2021) and discussed well-known deep learning architectures. The paper highlights the importance of automated disease detection in agriculture for early diagnosis and effective management. The key findings of their survey includes the use of deep learning models, such as convolutional neural networks (CNNs), for accurate disease identification, the significance of image preprocessing techniques, and the potential of transfer learning for improving classification performance in limited data scenarios. However, dataset limitations were not discussed very well.

**Table 1:** Top most cited papers in recent years.

Year	Reference	Datasets	Dataset limitations	Research limitations	Results Comparison
2019	H. Saleem (Saleem et al., 2019)	×	×	×	×
2019	S. Kaur (Kaur et al., 2019)	×	×	√	√
2021	Liu J (Liu and Wang, 2021).	√	√	√	×
2021	Ngugi L.C (Ngugi et al., 2021).	√	×	√	√
2019	Shruthi U (Shruthi et al., 2019).	×	×	×	√
2021	Dhaka V.S (Dhaka et al., 2021).	√	×	√	√
2021	Li L (Li et al., 2021).	√	×	√	√
2020	Hassan R.I (Hasan et al., 2020).	×	√	√	√
2020	Nagaraju M (Nagaraju and Chawla, 2020).	√	×	√	√
2020	Singh V (Singh et al., 2020).	×	×	×	
2020	Chouhan S.S (Chouhan et al., 2020).	×	×	×	×
–	Ours	√	√	√	√

**Table 2:** Most recent papers.

Year	Reference	Datasets	Dataset limitations	Research limitations	Results Comparison
2022	Jackulin C (Jackulin and Murugavalli, 2022).	√	×	×	√
2022	Ghosh D (Ghosh et al., 2022).	×	×	×	√
2022	Tugrul B (Tugrul et al., 2022).	√	√	√	√
2022	Altalak M (Altalak et al., 2022).	×	×	√	×
2022	Rokhman N (Derisma et al., 2022).	×	×	×	×
2022	Jhajaria K (Jhajharia and Mathur, 2022).	×	×	×	×

### 1.2 Our contributions

In this paper, we conducted a survey about conventional and latest application of deep learning in plant pathology. It covers several sections of deep learning technologies in Plant Pathology such as the use of conventional methods of image classification, Artificial Neural Networks (ANNs), Convolutional Neural Networks (CNNs), Vision Transformers and other techniques. We also discuss related concepts, applications and limitations involved in its implementation. The main contributions of the study is described as below:

• Present a comprehensive survey about prominent datasets used for plant disease identification and discuss their limitations
• Review Image capturing and processing techniques for preparing datasets
• Review conventional and deep learning techniques for the classification, detection and segmentation of diseases
• Show challenges and open research directions for the implementation of Plant Pathology using Deep Learning

**Figure 2:**
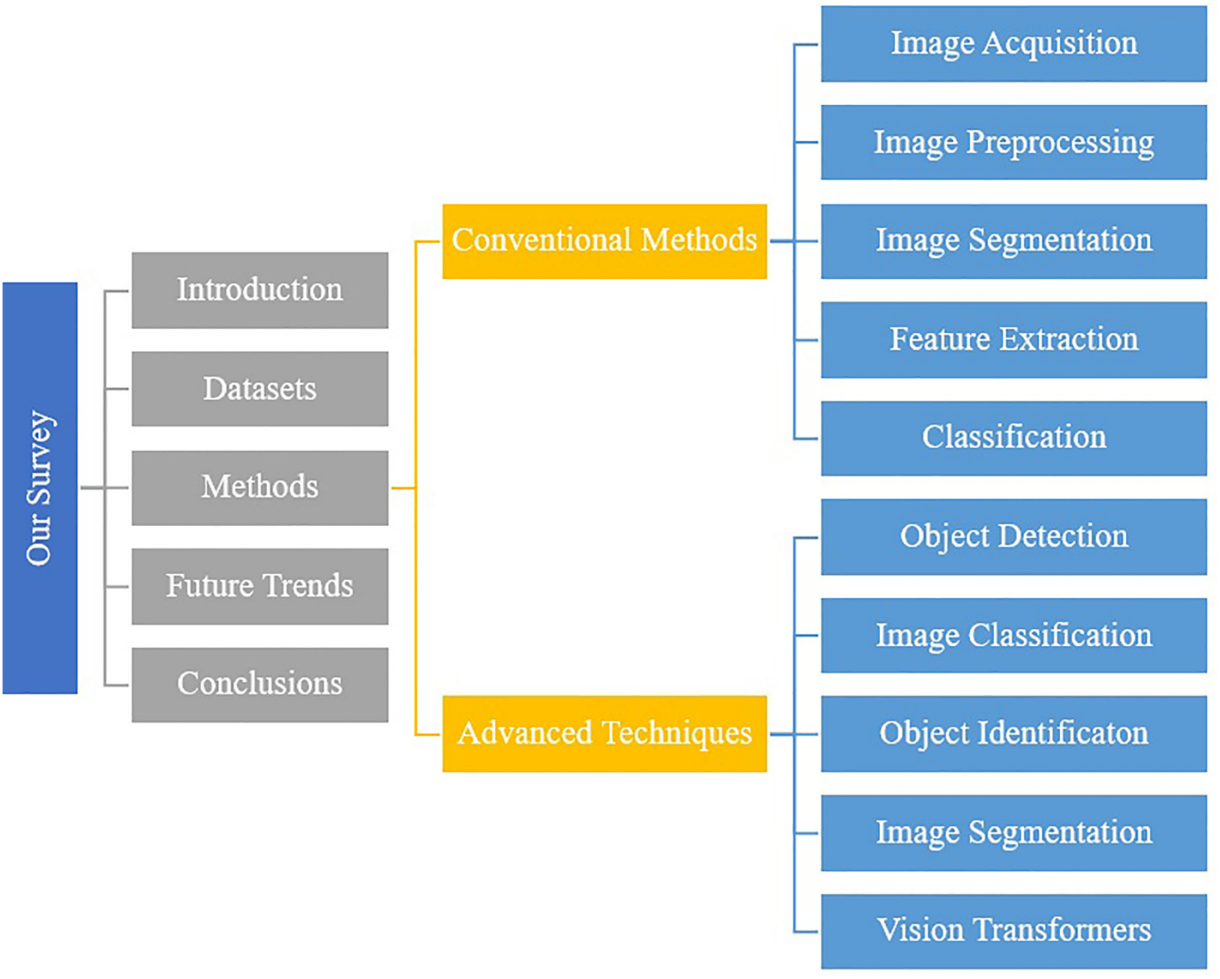
The structure and organization of the paper at a glance.

As shown in **Figure 2**, this survey is divided into four sections. In Section 2, we discuss datasets that have been used for plant pathology and their properties, image acquisitions, focus areas and discuss their limitations. In section 3, we described the methods that have been used by researchers, results and their limitations. Finally, in Section 4, we summarize and discuss future approaches.

## 2 Datasets

Datasets play a vital role in producing accurate results with deep learning models. A large number of images are required in a dataset for training deep learning models to classify diseases. It has been observed that the Plant Village dataset is the most popular publicly available dataset for researchers. However, researchers also opted for customized small datasets in the reviewed studies. These customized datasets were focused on classifying particular disease(s). A summary of datasets that are used by researchers in this survey has been presented in **Table 3**.

**Table 3:** Summary of existing relevant datasets.

Dataset	Environment	Plant Species	Disease Classes	No. of Images	Annotation Type
Plant Village	Lab-Controlled	14	26	54,309	Bounding Box
PlantDoc	Real-field	13	17	2,598	Bounding Box
Digipathos	Lab-Controlled	21	171	3,000	Bounding Box
PlantCLEF2022	Real Field	80K	–	4,000,000	Specie Labels
Rice Disease Image Dataset	Lab-Controlled	1	3	3,355	Bounding Box
Rice Leaf Disease Dataset	Lab-Controlled	1	3	120	Bounding Box

### 2.1 Plant village

The Plant Village dataset (Hughes and Salathé, 2015) was published in 2015 and it contains a total of 54,306 images depicting both healthy and infected leaves. Each image in the dataset is labeled with a unique identifier, plant species, and the disease or health status of the plant. The dataset is divided into predefined training and test subsets, encompassing 14 different crops that are further segregated into 38 classes. Notably, tomato is the most common species in the dataset, accounting for 43.4% of the images.

**Figure 3:**
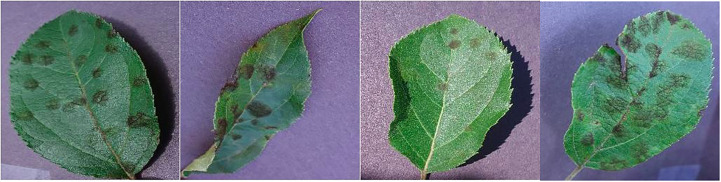
Sample images showcasing instances of Apple Scab disease within the Plant Village dataset.

**Figure 4:**
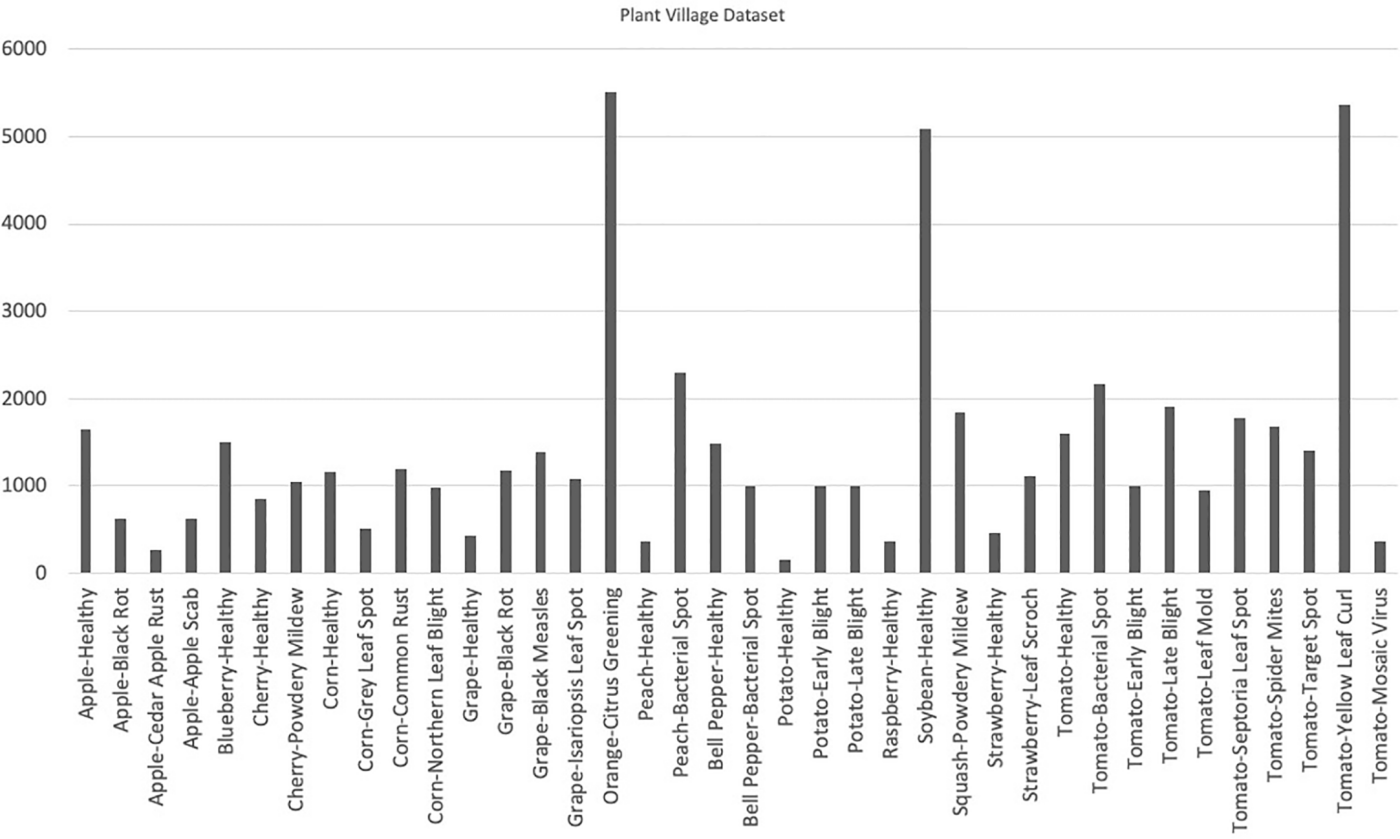
Visual representation of class distribution in the Plant Village dataset, revealing skewed proportions where certain classes dominate a significant portion while others occupy a smaller segment.

The Plant Village dataset offers a diverse range of plant diseases, with a total of 26 different diseases represented in the dataset. Early blight and late blight are the most common diseases in the dataset, accounting for 15.6% and 14.9% of the images, respectively. The availability of a large number of labeled images, coupled with the diversity of crops and diseases, makes the Plant Village dataset an important resource for researchers and developers working on plant disease detection models. With its well-defined training and test subsets, this dataset provides a robust means of training and evaluating machine learning models, and could ultimately lead to improved plant disease diagnosis and control in agricultural settings. The distribution of the number of images has been shown in **Figure 3**. **Figure 4** contains sample images of Apple Scab disease from the Plant Village dataset.

### 2.2 PlantDoc

**Figure 5:**
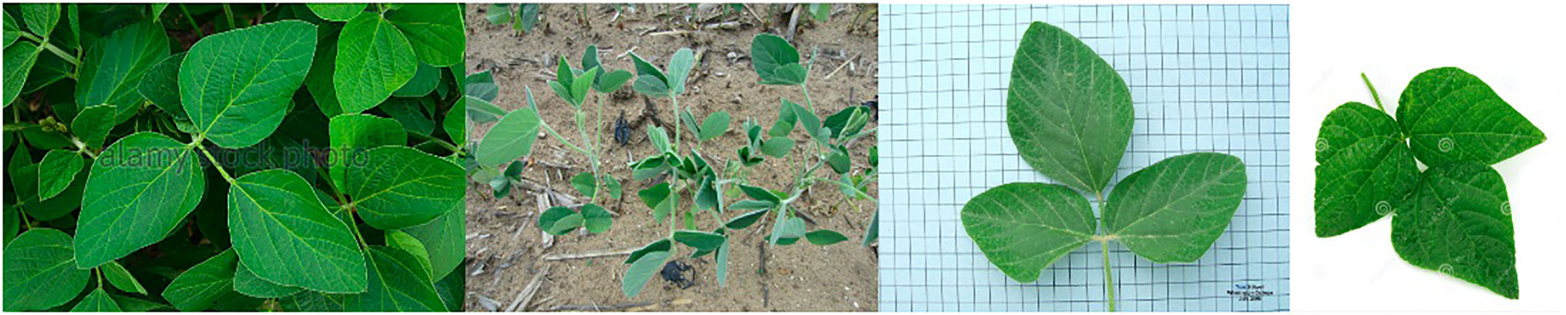
Sample images showcasing in-the-wild and lab-controlled instances of soybean within the PlantDoc dataset.

PlantDoc (Singh et al., 2020) dataset was created in 2019 and it is composed of 2,598 images depicting both healthy and infected leaves. This dataset was compiled from a variety of sources including images from Google and Ecosia and it features 13 different crops with 17 associated diseases. One notable characteristic of the PlantDoc dataset is that the images were captured under real-field conditions, providing a more realistic representation of the challenges faced by plant disease detection models. Despite its potential usefulness, the PlantDoc dataset has some limitations that must be considered. Due to the lack of domain expertise and knowledge, some of the images in the dataset are incorrectly classified, which impact the performance of machine learning models trained on this dataset. **Figure 5** contains sample images from the PlantDoc dataset.

### 2.3 Digipathos

**Figure 6:**
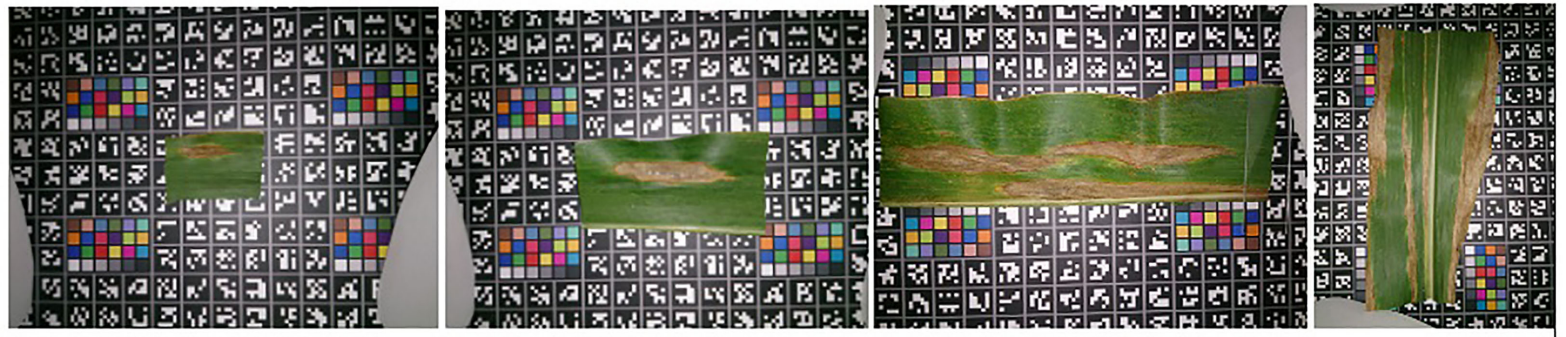
Sample images showcasing instances of Diplodia disease within the Digipathos dataset.

Digipathos dataset (Barbedo et al., 2018) consists of 3,000 images of cash crops namely rice, coffee, soybeans, beans, maize, wheat and other fruits and classifies 171 diseases among these classes. A major portion of images in this dataset is also acquired under a lab-controlled environment while a small portion contains real-field images. **Figure 6** contains sample images of Diplodia disease in Digipathos Dataset.

### 2.4 PlantCLEF2022

PlantCLEF2022 (Goëau et al., 2022) is an extensive dataset comprising over 4 million images and includes a wide range of 80,000 plant species. This dataset is compiled from two distinct sources; a trusted set built from the Global Biodiversity Information Facility (GBIF) and a noisy web dataset obtained from search engines like Google and Bing. In order to overcome balancing issues, the number of images are limited to a maximum of 100 per class with an average of 36.1 images per class. This large-scale dataset offers significant potential for the development and testing of machine learning models for plant species classification as it provides a diverse range of images that accurately represent the variation in plant species found in nature. The availability of a trusted set of images from the GBIF also ensures the reliability and accuracy of the dataset, making it a valuable resource for researchers.

### 2.5 Rice Disease Image dataset

**Figure 7:**
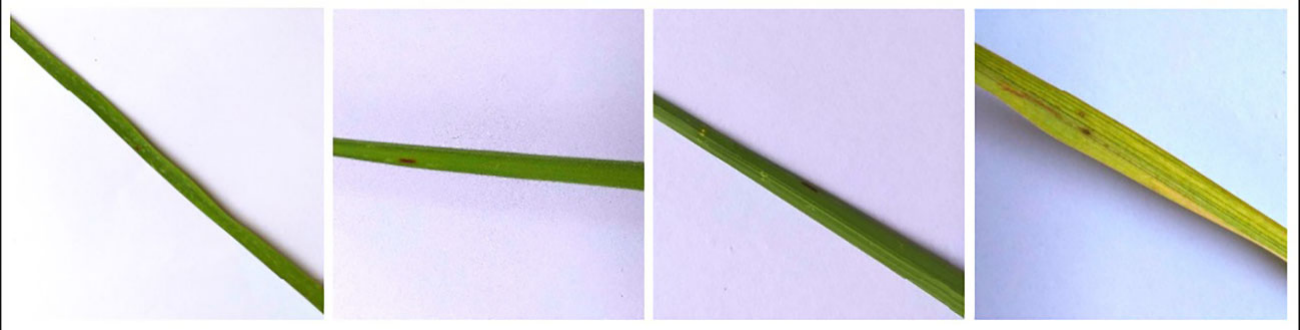
Sample images showcasing instances of Brown Spot disease within the rice disease dataset.

Rice disease dataset (Deng et al., 2021) contains 3,355 images of healthy and infected leaves of rice plants. These images are captured with white background and are not real-field condition images. The images in this dataset are divided into 3 disease classes such as Brown spot, Hispa and Leaf Blast. **Figure 7** contains sample images of Brown Spot diseased rice leaves in the Rice Disease dataset.

### 2.6 Rice Leaf Disease dataset

The Rice Leaf Disease Image dataset (Vbookshelf, 2019) is a compact yet well-balanced collection of 120 images of infected rice leaves. This dataset is composed of three distinct disease classes: Bacterial Leaf Disease, Brown Spot Disease, and LeafSmut with each class containing 40 images. Although small in size, it serves as a valuable resource for researchers in the field of rice disease detection, offering a diverse set of real-world images that can be used to train and evaluate machine learning models.

### 2.7 Discussion and limitations

Deep learning requires datasets with a large number of images to yield better accuracy. In order to increase performance, a deep learning model requires a large dataset with correct annotations. Due to limited expertise of an annotator, some of the infected areas of the leaf go unmarked in the image or they get wrongly annotated. This has been reported by the curators of the PlantDoc dataset (Singh et al., 2020) as well. Another issue encountered in annotating datasets is that certain diseases lack well-defined boundaries and the diseased tissue blends into the healthy tissues. Due to it, annotating boundaries of those diseases becomes difficult. Due to limited expertise and complex nature of these cases, the annotations may not accurately capture the extent of the disease. This adds to the complexity of dataset annotation and further impacts accuracy and learning of deep learning classifiers.

When working with datasets comprising real-field images, the issue of image illumination becomes a significant concern (Barbedo, 2016). Various factors related to image capture conditions, including specular lighting and overcast conditions, have a considerable impact on the visual attributes of the images. These conditions affect the way light interacts with the objects in the scene, resulting in variations in illumination. Consequently, datasets containing real-field images need to address the challenges arising from these illumination variations. The presence of specular lighting can introduce intense highlights or reflections, distorting the appearance of objects. Similarly, overcast conditions can lead to diffuse and even lighting, which alters the overall image characteristics (Shoaib et al., 2022). Therefore, researchers and practitioners working with such datasets must consider these aspects to ensure accurate and reliable analysis and interpretation of the image data.

When working with datasets that consist of a number of classes, it is essential to include images from all classes in a well-balanced proportion. A balanced dataset ensures that each class is represented adequately, thereby minimizing bias and allowing for more accurate and comprehensive analysis. By incorporating images from all classes in a balanced manner, the dataset can capture the full spectrum of visual characteristics and variations present in the real world. This inclusivity enables the development and evaluation of models or algorithms that are robust and adaptable to diverse conditions. Additionally, a balanced dataset prevents the dominance of certain classes, ensuring that the model’s performance is not skewed towards specific categories. A balanced dataset enhances the robustness of models, enables accurate analysis, and reduces bias by capturing the diverse visual characteristics present in the real world. Plant Village is a fairly big dataset but it has been observed in **Figure 3**. that this dataset is imbalanced i.e., it contains skewed class proportions where some classes take up a major portion of a dataset while a minor portion consists of other classes. This impacts the ability of deep learning models to learn significant features that distinguish a particular class from other classes (Ahmad et al., 2021). This lack of diversity in the Plant Village dataset results in over-fitting of data while learning (Ahmad et al., 2021). Other datasets such as Digipathos and Rice Disease Dataset contain very small amount of images which focus on particular diseases in certain plant species. This issue is addressed by Karam C (Karam et al., 2023). by proposing a novel GAN-based pipeline for data augmentation. This increases a small dataset size four-fold and enhances the performance of the lightweight object detection model by more than 38% points.

In a separate study (Barbedo et al., 2018), J. Barbedo emphasized the immense challenge associated with creating a comprehensive database for classifying plant diseases. This challenge primarily stems from the requirement of amassing a large and accurately annotated image collection encompassing all diseases related to plants. Annotating such datasets is an arduous task that demands significant labor and meticulous attention to detail. The process involves carefully labeling each image with precise information regarding the specific disease it represents. Due to the vast diversity of plant diseases and the complexity of their visual manifestations, achieving accurate annotations becomes crucial for training reliable disease classification models. Thus, the development of a comprehensive database for plant disease classification necessitates dedicated efforts in acquiring and meticulously annotating a wide range of images depicting various plant-related diseases.

In conclusion, deep learning models require large and accurately annotated datasets to achieve better performance and accuracy. However, the annotation process is challenging due to limited expertise, leading to missed or incorrect annotations of infected areas in images. Additionally, some diseases lack well-defined boundaries, making boundary annotation difficult. These challenges in dataset annotation further impact the accuracy and learning of deep learning classifiers. Moreover, when working with datasets containing multiple classes, it is crucial to ensure a balanced representation of all classes to minimize bias and enable comprehensive analysis. Imbalanced datasets, such as the Plant Village dataset, can lead to overfitting and hinder the model’s ability to learn distinguishing features. To address dataset limitations, innovative approaches like GAN-based data augmentation have been proposed, increasing dataset size and improving model performance. Creating a comprehensive database for plant disease classification requires dedicated efforts in acquiring and meticulously annotating a wide range of images depicting various plant-related diseases.

## 3 Methods

Plant disease identification methods are classified into 1) Conventional (Hand-Crafted features) and 2) Deep learning based methods.

### 3.1 Conventional methods

Conventional methods of object detection rely on hand-designed features such as Haar-like features (Zaidi et al., 2022), and Histogram Of Gradients (HOG) (Dalal and Triggs, 2005) and SIFT (Piccinini et al., 2012). Before extracting features, it is crucial to find the location or region, therefore, region selection methods are employed first to identify the regions with objects. It is also challenging because the same object can have different scales in images and could be at any location in the image. Therefore, whole image is inspected using a sliding window method and feature extraction techniques are applied on these regions before forwarding them to later stages.

Previously, it was very challenging to design a global feature extractor which can work efficiently and accurately for all types of objects. Mostly, features were designed for specific object categories, for instance, HOG features were designed for human detection. Finally, after the feature extraction, these features are fed to some classifier such as Support Vector Machines (SVM) (Malisiewicz et al., 2011), or AdaBoost (Freund and Schapire, 1997) to localize objects and assign them appropriate class categories in the image. Support Vector Machines (SVM) are powerful classifiers known for their ability to handle high-dimensional data and complex decision boundaries. They can effectively separate data points using a hyperplane and are less prone to overfitting. SVMs perform well in scenarios with limited training data and can handle large feature spaces. However, SVMs can be computationally expensive, especially when dealing with large datasets and may struggle with noise and outliers. On the other hand, AdaBoost(Adaptive Boosting) is an ensemble learning method that combines multiple weak classifiers to create a strong classifier. It is particularly effective in handling complex datasets with overlapping classes. AdaBoost can focus on misclassified instances and iteratively improve classification accuracy. It is relatively simple to implement and less prone to overfitting. However, AdaBoost can be sensitive to noisy data and outliers, and its performance heavily depends on the quality and diversity of the weak classifiers used.

**Figure 8:**
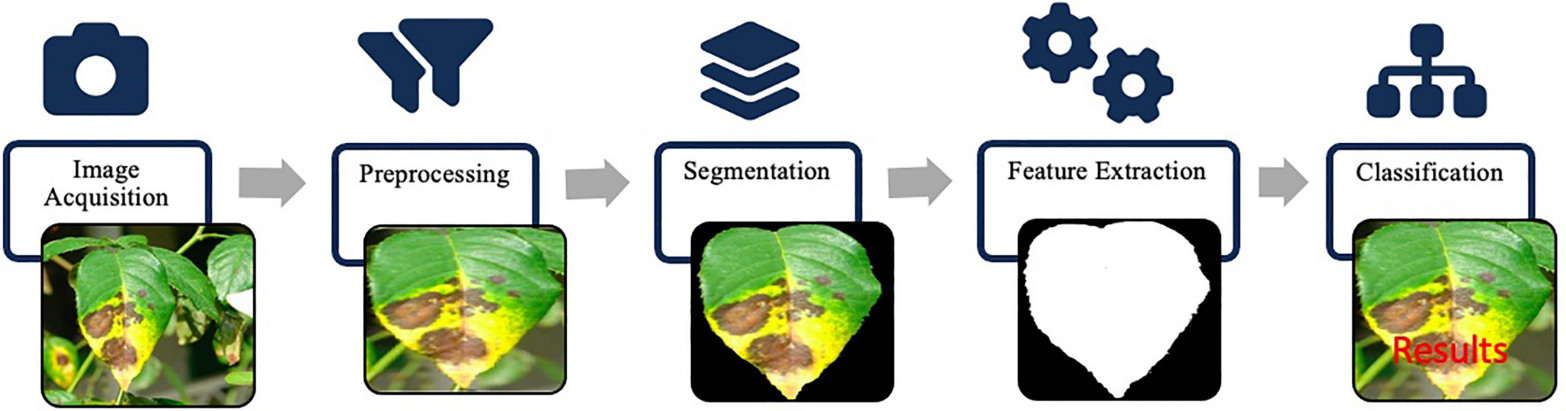
Hand-crafted features framework: image acquisition captures the input. Preprocessing enhances image quality. Segmentation identifies regions of interest. Feature extraction algorithms extract descriptive hand-crafted features. Classification utilizes these features for labeling and predictions.

Conventional methods require tremendous human effort and engineering to build a powerful object detection system. Furthermore, the region extraction methods also take huge computations because there are no region proposal mechanisms. Instead, regions are extracted from the whole image using the sliding window as described earlier. Such an approach lacks scalability and responsiveness, hence limiting the applicability in various scenarios where real-time processing is crucial. Moreover, these hand-designed features suffered from various commonly existing variations in images, such as illumination variation, and object pose variations. Most of the disease identification methods based on hand-crafted features consist of some common processing stages as shown in **Figure 8**. The identification process begins with acquiring digital images *via* image capturing device. Images are then pre-processed using image transformation, resizing and filtering etc. Then images are segmented using a suitable segmentation technique such as clustering, edge detection, region growing to extract the infected part of a plant. Later, features of interest such as color, shape or texture are extracted *via* feature extraction techniques. After that, classifiers are used to classify the images according to a specific problem.

#### 3.1.1 Image acquisition

Most of publicly available datasets (**Table 3**), contain images that are acquired using hand-held devices such as cameras. For the most of articles surveyed, camera has been used for acquiring images and creating databases. However, drones and Unmanned aerial vehicle (UAV) have also been used to capture aerial images of maize and weed in soybean plants by Ferreira D. S (dos Santos Ferreira et al., 2017). and Stewart E. L (Stewart et al., 2019). respectively. Lowe A (Lowe et al., 2017). used hyperspectral imaging to capture images which allowed them to capture wavelength beyond the limited range of human vision.

#### 3.1.2 Image preprocessing

**Figure 9:**
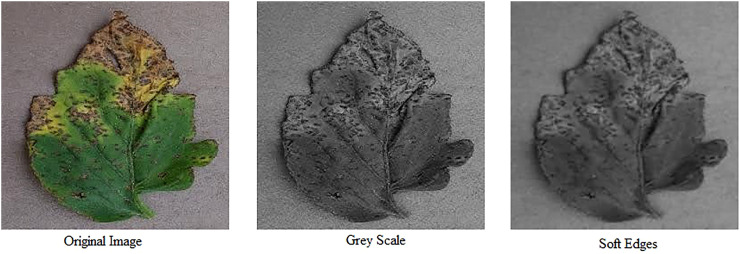
Image preprocessing: original image, greyscale conversion and soft-edged representation (Left to right).

Acquired images are preprocessed to highlight the area of interest i.e. diseased area of a plant. This involves image resizing (Mokhtar et al., 2015; Zhang et al., 2018; Hang et al., 2019; Militante et al., 2019), colorspace conversion (Al-Hiary et al., 2011; Mokhtar et al., 2015; Brahimi et al., 2017) and applying filters to reduce noise in an image to yield better outcomes while segmenting an image. **Figure 9** shows results of Grey Scaling and Soft-Edging against an original image.

#### 3.1.3 Image segmentation

**Figure 10:**
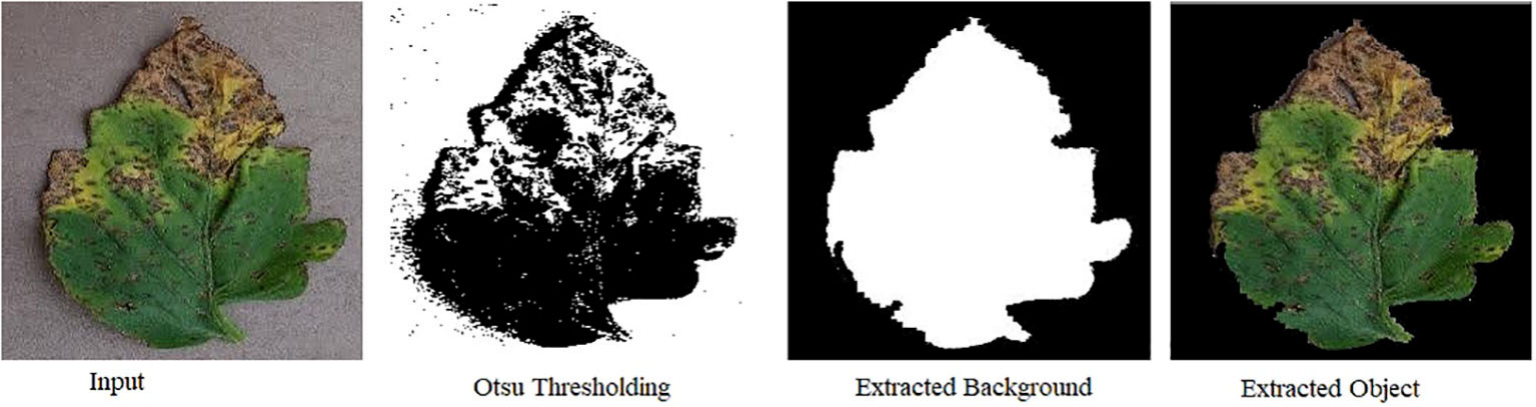
Visual depiction of image segmentation techniques: Otsu thresholding, background extraction, and foreground/object extraction, showcasing the distinct results achieved through each method.

Image segmentation is the process of categorizing an image into different regions based on the characteristics of pixels to identify objects or boundaries. It is the first step of most image-based tools for leaf analysis in which leaf is isolated from the background. There are several techniques that are used for image segmentation such as K-Means, Otsu thresholding, color-space conversions etc. In K-Means clustering, similar data points are grouped together while Otsu thresholding determines an optimal threshold for separating background and Object. Color-space conversions, edge detection and region growing are some of the techniques that are used for segmenting an image. **Figure 10** shows results of image segmentation techniques such as Otsu Thresholding, Background Extraction and Extraction of foreground or object.

#### 3.1.4 Feature extraction

Feature extraction is the process of extracting properties of a leaf such as its shape, size, texture, edges and color etc (dos Santos Ferreira et al., 2017). performed the feature extraction from each segment of the dataset using a collection of shape, color, texture and image orientation extractors implemented in common image processing toolboxes and libraries such as MATLAB Image Processing Toolbox, OpenCV, and Dlib (Cope et al., 2012). presented a review on image processing methods that have been applied in recent years to analyze leaf shape, venation, leaf margin features, leaf texture. Feature extraction is used in conventional object recognition. However, for deep learning, feature extraction is not required since deep learning models generates these features themselves. Islam M (Islam et al., 2017). has used image segmentation with multiclass SVM on RGB based features of 300 images acquired from Plant Village dataset and achieved an accuracy of 95% to classify 2 diseases; late blight and early blight in potato leaves.

#### 3.1.5 Classification

Classification is the process of analyzing image features. It classifies the image data into categories. This process is categorized into supervised, unsupervised and semi-supervised classification. Supervised classification is a machine learning paradigm that deals with data available in the form of labelled examples. In supervised learning, training data consists of input values and desired output values. While in unsupervised learning, algorithm figures out patterns from unlabeled data. Some of the popular classification techniques are Logistic Regression, K-nearest neighbor, support vector machine (SVM) and artificial neural networks. SVM were implemented for sugar beet disease (Rumpf et al., 2010) and depending upon severity of disease, classification accuracy of 65% was achieved when 1-2% area of the leaf was diseased and accuracy increased to 90% when diseased area of the leaf was 10%-15%. Pattanaik A. P (Pattanaik et al., 2022). proposed an approach where late blight disease was detected using “Improving Localization and Classification with Attention Consistent Network” (ILCAN) approach and achieved 98.9% accuracy which was better than the accuracy of 91.43% achieved by Grad-CAM++ (Chattopadhay et al., 2018).

### 3.2 Detection and Identification using advanced techniques

Artificial neural network (ANN) involves a collection of connected units called neurons. ANN is composed of 3 types of layers which contains these neurons named as input, hidden and output layer. The design of ANN is inspired by that of a biological brain. Like a brain, a neuron in ANN receives an input, processes it and outputs it to the neurons of the next layer. Advancements in computing power enabled to design deeper ANNs especially neural networks based on convolutional layers called convolutional neural networks (CNNs). The convolutional layer’s parameters contains a set of learnable filters called kernels. A CNN consists of four layers i.e. input, convolutional and pooling, fully connected layer, and output layer. Similarly, these type of approaches were applied to plant disease classifications. Mohanty S. P (Mohanty et al., 2016). used CNNs to classify 26 disease in 14 crops and achieved an accuracy of 99.35% on testing data. However, their accuracy dropped to 31% when classifier was tested on images that were different from the training dataset.

#### 3.2.1 Object detection based on deep learning

Object detection deals with the classification as well as localization of an object in an image. Object localization is the identification of an object in an image and drawing a bounding box around it. In deep learning, object detection is achieved using supervised learning by providing annotated images. Convolutional neural networks (CNNs) are used for object detection due to their property of high feature representation. Two main types of object detectors are 1) Two-stage object detectors and 2) One-stage object detectors. Two-stage detectors provide high localization and recognition accuracy whereas one-stage detectors have high inference speed.

In two-stage object detectors, detection is divided into two stages. First stage deals with the localization of an object and the second stage deals with the classification of the object that has been localized. Object localization is the identification of an object and then drawing a bounding box around it. While two stage object detectors provide a high accuracy in detection, it comes with a trade-off of slow detection speed. Some of the most popular CNNs include Region-based Convolutional Neural Network (R-CNN) (Altalak et al., 2022), Faster R-CNN (Ren et al., 2015) and Mask R-CNN (He et al., 2017).

**Figure 11:**
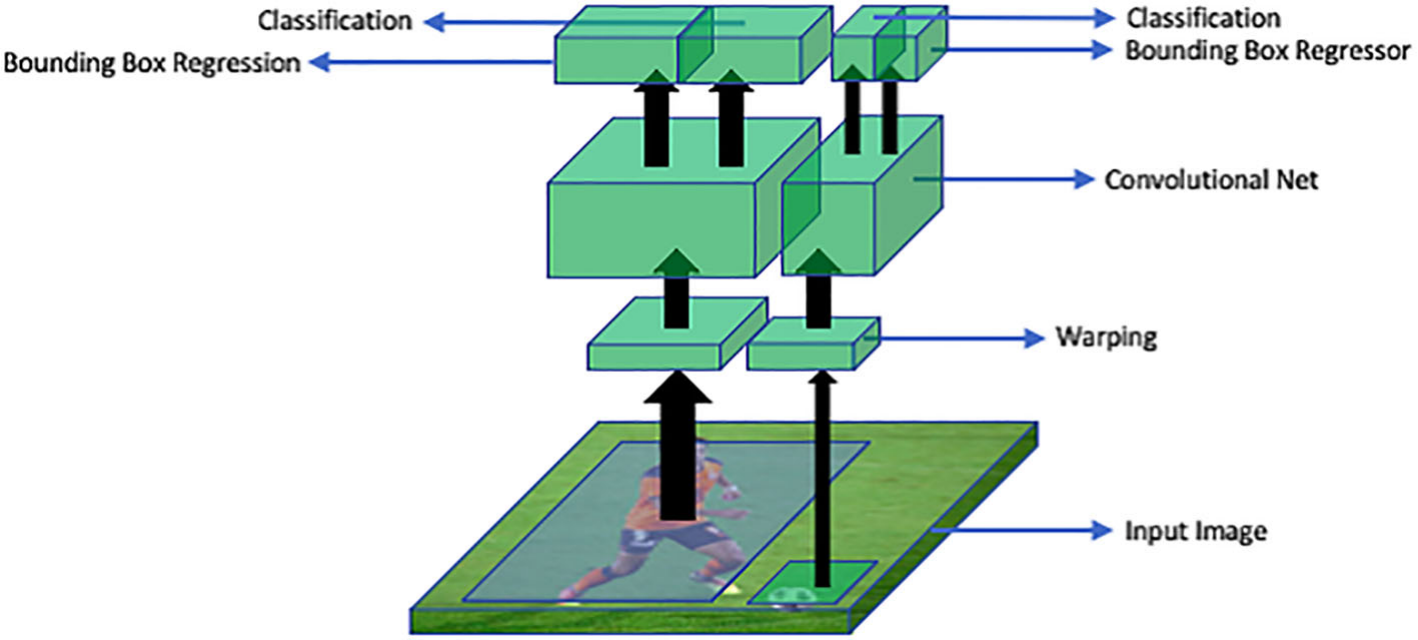
An overview of R-CNN architecture: RoI warping extracts regions of interest (RoIs) from the input image. A convolutional neural network (CNN) processes these RoIs to extract features. The bounding box regressor refines object locations and sizes. Classification assigns labels to the objects based on the extracted features.

R-CNN deep learning method for detection of objects proposed by Ross G (Altalak et al., 2022). In R-CNN, region proposals or regions of interest are extracted using a selective search algorithm. Selective search algorithm groups regions together based on their pixel intensities. Then these regions are re-scaled into the input image size and features from each candidate region are extracted with the help of convolutional neural networks. Then a support vector machine (SVM) classifier is used to detect the presence of an object within the extracted region. Then, output is generated in the form of a bounding box using a linear regression model. This process has been explained in **Figure 11**.

**Figure 12:**
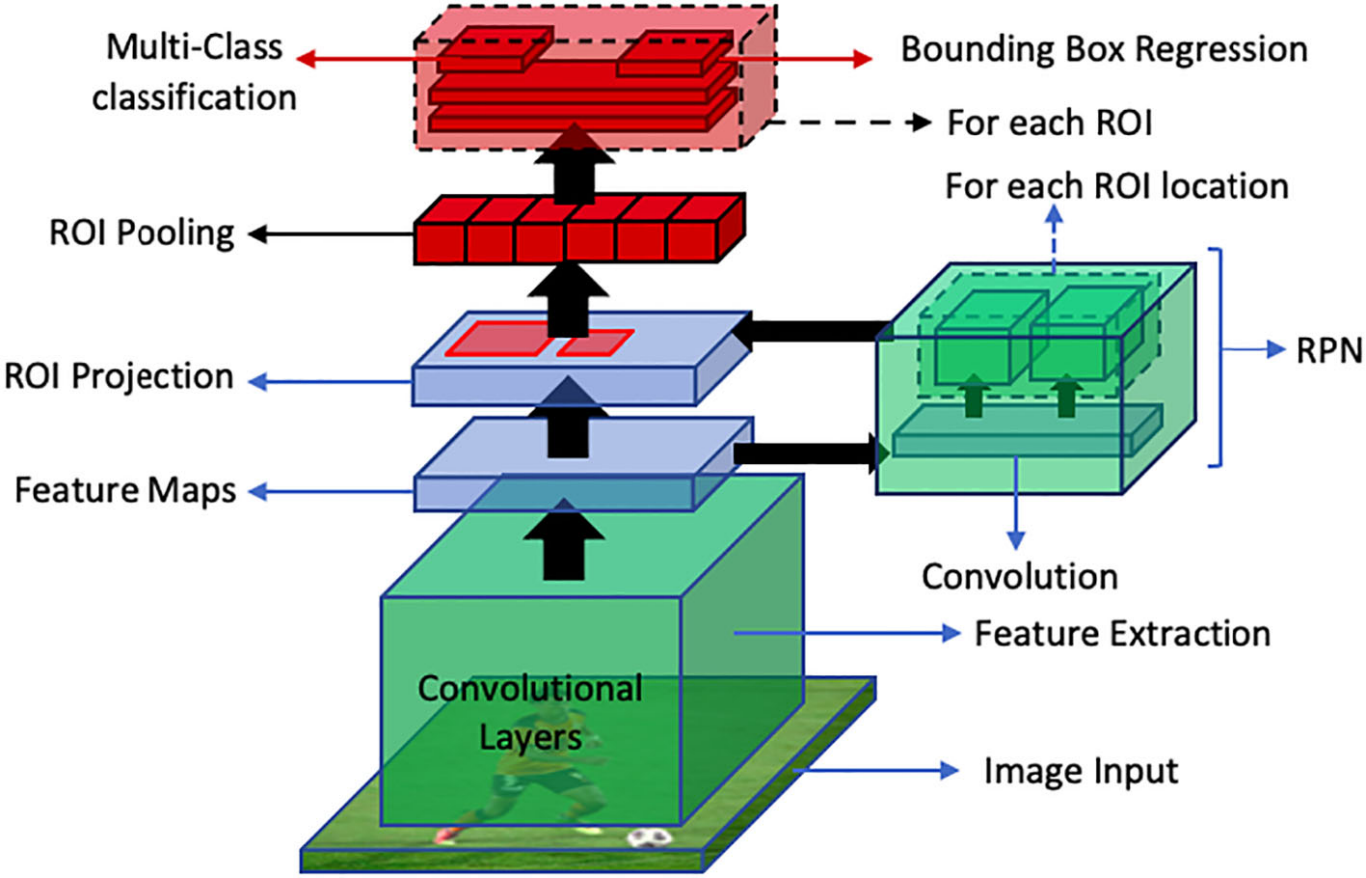
An overview of faster R-CNN object detection pipeline. The RPN generates region proposals, while ROI Projection maps these proposals to feature maps. Finally, ROI Pooling extracts fixed-length feature vectors for classification and bounding box regression for object detection.

Faster R-CNN is an end-to-end deep learning detector which replaces region proposal algorithms such as selective search, multiscale combinatorial grouping or edge boxes with CNN called Region Proposal Network (RPN). This improves the detection speed of Fast R-CNN. In this approach, an image is fed into the CNN which produces a feature map. This feature map is then provided as an input to the RPN which provides multiple object proposals using a sliding window on the provided feature maps. In the sliding window, the network generates reference boxes of different dimensions. Class-specific features are selected from these reference boxes. For each reference box RPN predicts the objectiveness probability and bounding box regressor to adjust the box to fit the object. RPN then returns multiple object proposals along with their objectiveness score. Then the ROI pooling layer is applied to extracted object proposals to transform them into a fixed dimension. The feature vectors are then fed into a fully connected layer including a softmax layer for categorization and linear regression layer for bounding box generation. This process has been illustrated in **Figure 12**.

Mask R-CNN (He et al., 2017) deals with image instance segmentation and is based on the R-CNN family of networks, which are a popular object detection method. R-CNN performs a pixel-level segmentation and decides the probability of it being a part of an object. Like Faster R-CNN, it also uses RPN but it is differentiated by its three outputs for individual object proposals which include a bounding box offset, a class label, and the object mask. Mask R-CNN uses an RoIAlign layer instead of a RoI pooling layer to preserve spatial information and avoid misalignment in the RoI pooling layer resulting in increase in its detection accuracy. RoIAlign layer uses binary interpolation for feature map creation and evaluates feature values at each sampling point.

Object detection itself is a complicated problem as it contains various components and engineering. These components rely on image classification networks as backbones. In other words, these backbones act as the feature extraction network for the object detection framework. Some of the most prominent backbone networks include VGG (Simonyan and Zisserman, 2014), EfficientNet (Tan and Le, 2019), MobileNet (Howard et al., 2017), Inception (Szegedy et al., 2015) and ResNet (He et al., 2016).

VGGN is a CNN based architecture which is similar to AlexNet but expands its depth to 16-19 layers. Initially, the success in deep learning was associated with the depth of the network i.e., increasing the number of layers tends to increase the performance. Mainly, the constraint was the availability of computation power. VGG solved this problem by reducing the kernel size. Previously, using a kernel size of 7 x 7 was the norm. Authors of VGG showed that having three layers with 3 x 3 kernels has the same exposure as that of 7 x 7 with only half the trainable parameters. Moreover, having 3 layers meant it had 3 more non-linear activations as compared to one layer with kernel size of 7 x 7. Overall, VGG uses the kernel size of 3 x 3 and pooling kernel of size 2x2 which significantly improves its performance while having a deeper network structure. VGG16 and VGG19 are the most popular variations having 16 and 19 layers respectively. Specifically, VGG16 contains 13 convolutional layers and 3 fully connected layers and has an extensive network of about 138 million parameters.

The bottleneck in the performance gain of CNNs was the computational requirements. InceptionNet (Szegedy et al., 2015) addresses this issue by reducing the computational complexity by introducing the 1 x 1 convolutional layers. Hence, increasing the extent to which the network’s depth can be increased. Moreover, InceptionNet won the prestigious ILSVRC challenge in 2014, which is an image classification benchmark based on ImageNet (Deng et al., 2009) dataset having 1000 classes. Furthermore, InceptionNet is built upon the basic Inception module which contains parallel layers having 1 x 1, 3 x 3, and 5 x 5 kernel sizes, therefore having the capability to capture information with different spatial exposures simultaneously. InceptionNet was further improved by introducing the concept of batch normalization which improved the training times by InceptionV2 (Ioffe and Szegedy, 2015). The most characteristic feature of InceptionNet is its huge depth as compared to the predecessors while having only a partial number of trainable parameters i.e., 6.7 Millions.

EfficientNet (Tan and Le, 2019) is a CNN architecture which was introduced in 2019, that achieves high performance while being computationally efficient. It uses compound scaling to balance accuracy and efficiency by scaling the depth, width, and resolution of the network. As compared to EfficientNet, MobileNet (Howard et al., 2017) is a lightweight CNN architecture designed for mobile and embedded vision applications. It reduces computations using depth-wise separable convolutions, achieving a good balance between accuracy and efficiency for resource-constrained devices.

**Figure 13:**
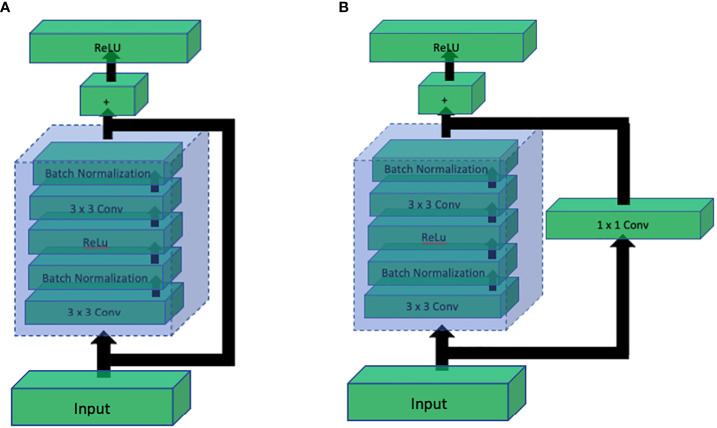
Comparison of ResNet residual blocks: **(A)** Residual block without 1x1 convolution, and **(B)** Residual block with 1x1 convolution. The addition of the 1x1 convolution in **(B)** enhances the representation power and allows the network to learn more complex features, leading to improved performance in deep learning tasks.

Up until the success of InceptionNet, it was known that increasing the depth of CNN architectures has a directly proportional relation with the performance. However, it was shown by Kaiming He et al. (2016) that this direct relation is not linear, and the performance starts getting saturated after increasing the depth to some extent. If the network depth is further increased, the performance starts decreasing. They further showed that the major cause of drastic performance degradation was vanishing gradients. This means that as the network depth increases, it gets more challenging to back propagate the error through the large number of layers where the gradient ultimately vanishes, and the weights of the layers stop updating according to the calculated error. To solve this problem while being able to build deeper networks, they proposed a simple yet clever way to preserve the original information by introducing the concept of skip connections. Skip connections introduced a parallel path that bypasses the convolution block and is added again with the output of the convolution block as shown in **Figure 13**. This simple technique helped construct ResNet architecture with depth of 101 and even 152.

#### 3.2.2 Disease identification based on Image classification

As discussed earlier, image classification is a fundamental task in computer vision. Therefore, a number of methods have utilized image classification for plant disease identification as shown in **Table 4**.

**Table 4:** Comparison of image classification models and results.

Dataset	Year	Model	Accuracy	Subject	Disease Classes
Plant Village	2016	AlexNet, GoogleNet, CNN	99.35%	14 Crops	26
	2018	VGG	99.53%	25 Plants	38
	2017	Multiclass SVM	95%	Potato	2
	2017	GoogleNet	99.18%	Tomato plant	9
	2017	GoogleNet	98.6%	Banana Leaf	2
Custom	2022	SE-ResNet-101, ILCAN	98.99%	Late Blight Detection	1
	2020	InceptionV3, VGG16, VGG19	93.4%	Tomato Leaves	6
	2020	CNN	98.4%	Corn	2
	2019	CNN	96.5%	Leaf images	11
	2019	VGG16 with Inception and Squeeze-and-Excite Module	91.7%	Apple and Cherry	4
	2019	CNN	98.8%	Maize Leaves	8
	2018	GoogleNet	98.9%	Maize Leaves	8
	2016	CaffeNet, CNN	96.3%	Leaf images	13

Ahmad (Ahmad et al., 2020) used VGG16, VGG19, ResNet and InceptionV3 (Szegedy et al., 2016) and fine-tuned the network to get optimal results on tomato leaves dataset containing images of both types; lab-controlled and real-field, and classified 6 disease classes. As per their results, InceptionV3 yielded an accuracy of 99.60% on lab-controlled images and 93.70% on images captured in-the-wild.

Mishra S (Mishra et al., 2020). proposed a deep learning based approach for disease recognition in corn plants on a stand-alone device such as a smartphone or Raspberry pi. They trained their model on Intel Movidius system chip and were able to achieve an average accuracy of 98.4%.

Militante (Militante et al., 2019) used CNN on plant village dataset to yield an average accuracy of 96.5% on classification of 4 types of grape leaf diseases, 4 types of corn leaf disease, 4 types of apple leaf disease, 6 types of sugarcane diseases and 9 types of tomato leaf diseases. The dataset consisted of 35,000 images from plant village and testing was done on 1,000 images taken in-the-wild.

Hang J (Hang et al., 2019). used VGG16 with inception and Squeeze-and-Excite Module to classify 4 diseases in apple, cherry and corn and yielded an accuracy of 91.7% by generalizing the AI Challenger dataset.

Zhang X (Zhang et al., 2018). were able to achieve 98.9% and 98.8% accuracy using improved deep neural network architectures; GoogLeNet (also known as InceptionV1) (Szegedy et al., 2015) and Cifar10 respectively. They reported the possibility to improve recognition accuracy by adding Relu function, increasing diversity of pooling operations and including adjustments to the model parameters.

Ferentinos (2018) used AlexNet (Krizhevsky et al., 2017), AlexNetOWTBn (Krizhevsky, 2014), GoogLeNet and VGG on the Plant Village dataset and yielded an accuracy 99.53% on classifying 38 classes in 25 different plant species.

#### 3.2.3 Disease identification based on object detection

Object detection encompasses both the categorization and positioning of an object within an image. Localization refers to the process of recognizing an object in an image and delineating it with a bounding box. In deep learning, object detection is accomplished through supervised learning, where annotated images serve as training data. R-CNN (Girshick et al., 2014), Fast R-CNN (Girshick, 2015), Faster R-CNN (Ren et al., 2015), YOLO (Redmon et al., 2016) and Single shot MultiBox Detector(SSD) (Liu et al., 2016) are the most popular CNN based object detection algorithms. Object detection has been used in plant disease identification to detect presence of a disease in the provided image of a plant. Faster R-CNN were used with VGG-Net and ResNet for identification of pests and tomato diseases and mAP of 85.98% was achieved to classify 9 categories by Fuentes A (Fuentes et al., 2017). Similarly, Ozguven M (Ozguven and Adem, 2019). proposed a method for detection of beet leaf spot disease by optimizing parameters of CNN and classification rate of 95.48% was achieved. Zhou G (Zhou et al., 2019). proposed a method for fast detection of rice blast, bacterial blight using fusion of FCM-KM and Faster R-CNN to achieve an accuracy rate of 97.2%. Ina latest research, Xie X (Xie et al., 2020). implemented Faster R-CNN on the grape leaf disease dataset by utilizing InceptionV1, ResNet V2 and achieved accuracy of 81.1%. **Table 5** provides a concise overview of various object detection methods and their corresponding results.

**Table 5:** Object detection methods and results.

Year	Model	Disease Classes	Results
2017	Faster R-CNN, VGG, ResNet	9	85.98% mAP
2019	Faster R-CNN	1	95.48% Accuracy
2019	Faster R-CNN, FCM-KM	–	97.2% Accuracy
2020	Fast R-CNN, InceptionV1, ResNet V2	4	81.1% Accuracy

#### 3.2.4 Disease identification based on image segmentation

**Figure 14:**
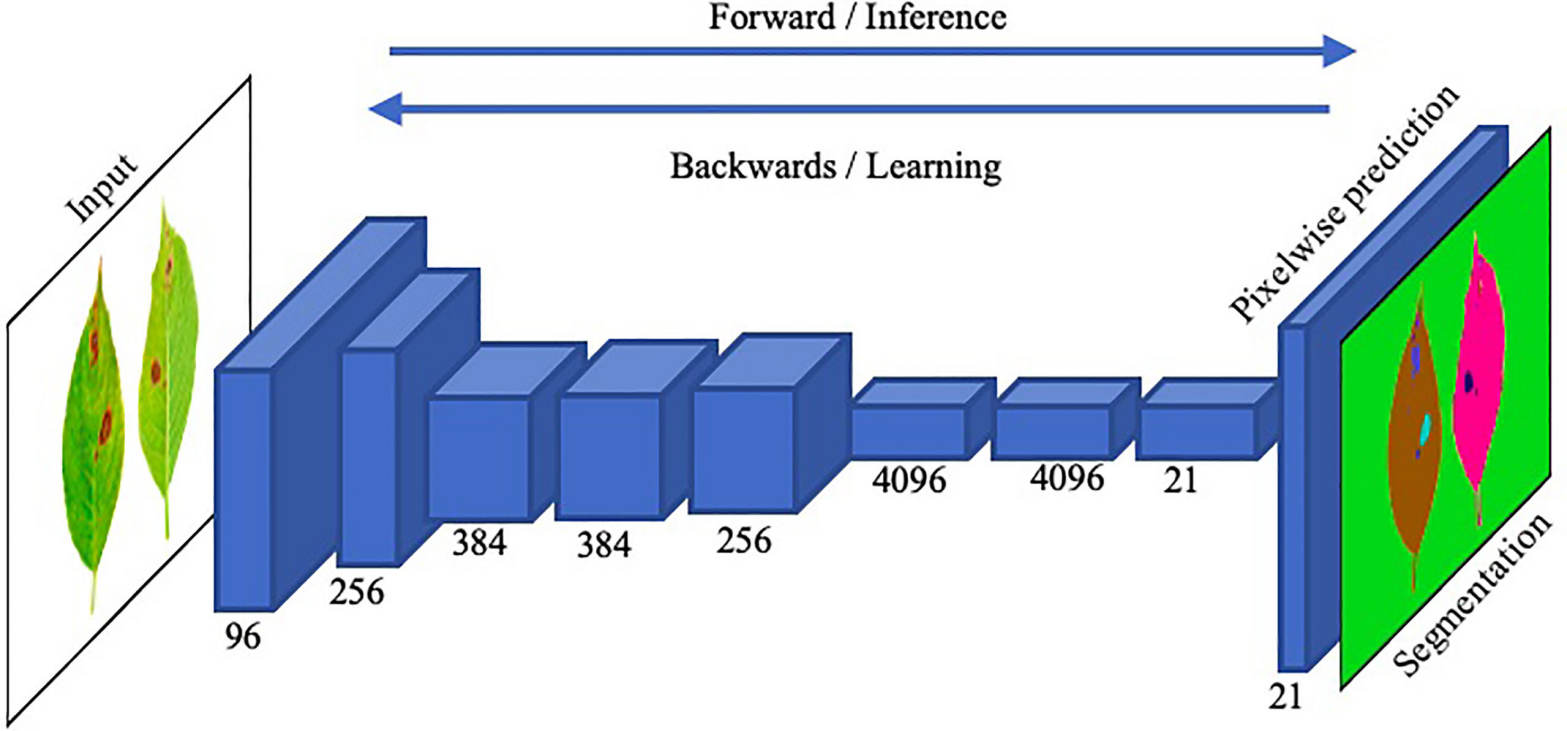
An illustration of the functionality of a straightforward Fully Convolutional Network (FCN) for precise image segmentation.

Image segmentation in plant disease diagnostics is the categorization of semantic and instance segmentation of diseased and healthy area. It not only provides details of location and category of the segmented region but also provides properties such as area, length and outlines. A summary of image based segmentation studies is shown in **Table 6**. Image segmentation is further divided into two main architectures namely Fully Convolutional Networks (FCN) and Mask R-CNN. FCN deals with implementation of locally connected layers only. This excludes the dense layer which results in less trainable parameters which lead to faster training of the network. A simple Fully Convolution Network for Image Segmentation has been shown in **Figure 14**. To compensate the lost information during down sampling, skip connections are also utilized in some FCNs e.g., U-Net (Ronneberger et al., 2015). U-Net is an encoder-decoder structure which introduces a layer-hopping connection and fuses the feature map from one encoder’s layer to its corresponding decoder layer. A modified version of U-Net was utilized by Lin K (Lin et al., 2019). to segment cucumber powdery mildew leaves to achieve an average accuracy of 96.08%.

**Table 6:** Image segmentation methods and results.

Year	Model	Segmentation Subject	Results
2019	Modified U-Net	Cucumber powdery mildew	96.08% Accuracy
2019	Mask R-CNN	Northern Leaf Blight	96% Accuracy
2019	Mask R-CNN, ResNet-101	Tomato Diseases	99.64% mAP

Mask R-CNN deals with image instance segmentation and is based on R-CNN family of networks, which are popular object detection methods. Mask R-CNN were implemented to individually segment diseased instances with accuracy of 96% by Stewart E. L (Stewart et al., 2019).. Mask R-CNN with object detection networks were utilized by Wang Q (Wang et al., 2019). to segment location and shape of diseased area of tomato disease and classify into 11 diseases with mAP of 99.64% using ResNet-101.

#### 3.2.5 Disease identification based on vision transformers

Transformers (Vaswani et al., 2017) were originally introduced for the Natural Language Processing (NLP) tasks such as language classification or language generation. The key idea of transformers is based on the attention mechanism. Specifically, it was proposed to compute the self-attention between the different word tokens in a sentence. The architecture of the Transformers itself is simple as it contains multiple Multi-Layer Perceptron (MLP) layers, so it is not wrong to say that the transformer network is essentially a mapping network that simultaneously computes all pairwise interactions among elements in an input sequence.

After the success of Transformers in the natural language domain, researchers from the vision domain have also attempted to apply it to several vision tasks such as for image classification, Vision Transformers (ViT) (Dosovitskiy et al., 2020) is the most prominent.

Even though Transformer architectures have shown impressive performance on various language tasks, it is challenging to apply to the vision domain mainly due to the high dimensional vision data. ViT proposed to solve this problem by dividing the input image into 16 × 16 patches and flatten them sequentially and the rest of the process is similar to the original Transformer architecture used for the language tasks. Essentially, the image is broken down into a sequence of patches, which is similar to having a sequence of language tokens so that the Transformer architecture can be easily adapted for image data.

**Figure 15:**
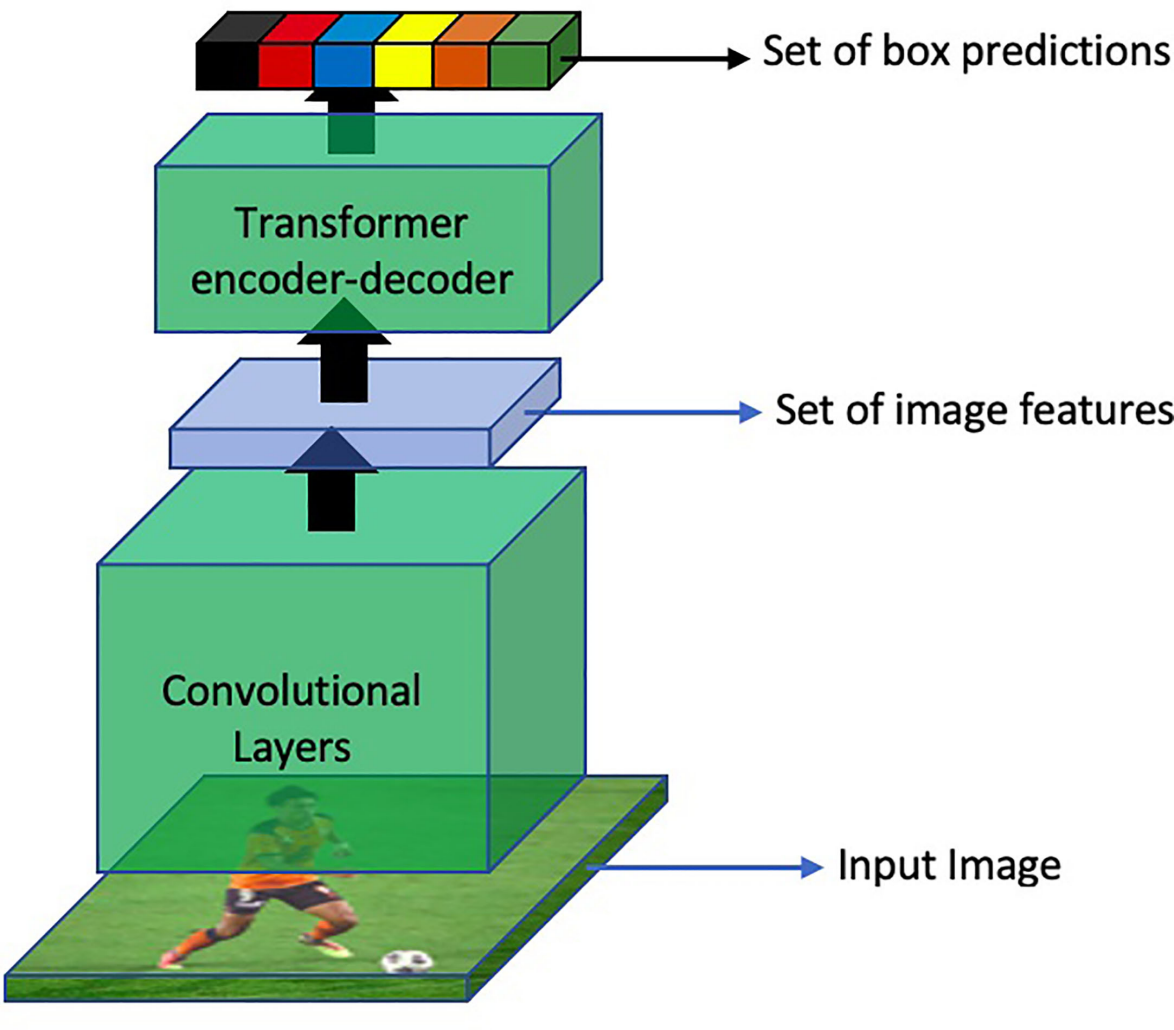
An overview of object detection based on DETR.

Following ViT, several other researchers utilized the power of these architectures for other vision tasks. In the specific domain of object detection, DETR (Carion et al., 2020) proposed an end-to-end object detection method using Transformers. The overall method of DETR is shown in **Figure 15**

Moreover, DETR models the object detection problem as a direct set prediction problem and follows the original encoder-decoder based architecture. Another interesting feature about DETR is that in contrast to traditional object detection models, DETR predicts all the objects in an image at once. These object predictions are then bipartite matched with the ground truth. Another revolutionary aspect of this approach is that it eliminates the need to hand-design object detection components which were previously a part of all object detection methods such as Region Proposal Network (RPN), Feature Pyramid Network (FPN), Non-Maximal Suppression (NMS), or Spatial Anchors.

Specifically, The architecture consists of a backbone convolutional neural network (CNN) encoder that extracts feature maps from the input image. These feature maps are then passed to a transformer-based decoder, which generates a set of fixed-size bounding boxes, their corresponding class labels, and a special label for no object detection. The decoder uses self-attention mechanisms to capture global context information and process the object queries, which represent the potential locations of objects in the image. It predicts the object class and regresses the bounding box coordinates for each query. To encourage accurate predictions, DETR utilizes bipartite matching with Hungarian algorithm (Kuhn, 1955) during training, aligning predicted and ground-truth boxes. It also incorporates positional encodings (Parmar et al., 2018; Bello et al., 2019) to maintain spatial information in the transformer architecture.

Mingle X (Xu et al., 2022). proposed a transfer learning approach to achieve plant disease detection through few-shot learning. To reduce computation cost, they have employed a dual transfer learning. Their Vision Transformer (ViT) model is first pre-trained using the ImageNet dataset in a self-supervised manner and then fine-tuned using the PlantCLEF2022 dataset in a supervised fashion. They name their approach dual transfer learning because the ViT-L model is trained with datasets and transferred twice. The ViT-L model comprises 24 transformer blocks, with a hidden size of 1024, an MLP size of 4096, and 16 heads for each multi-head attention layer, resulting in approximately 307 million trainable parameters. They are comparing their models with several other models with the same settings, most of which follow the fine-tuning schemes in Masked Auto Encoder (MAE). Their experimental results suggest that their approach surpasses other state-of-the-art CNN-based models and achieves more accuracy when trained on a smaller dataset. Specifically, their model achieves 44.28 mAcc as compared to the second best-performing RN50-IN which achieves 23.46 mAcc in a 1-shot case, and achieves 86.29 mAcc as compared to RN50-IN, which achieved 73.53 mAcc in a 20-shot case. It’s worth noticing that the gap between the accuracies gets shorter as the number of training images increases.

Similarly, Yasamin B. presents a novel approach in (Borhani et al., 2022) to real-time crop disease classification using a ViT (Vision Transformer) architecture. The proposed model utilizes lightweight ViT models to achieve comparable performance to convolutional-based models. The evaluation is performed on three datasets; Wheat Rust Classification dataset, Rice Leaf Disease dataset and Plant Village. Results indicate that the ViT-based model outperforms CNNs in terms of accuracy, while still achieving comparable performance. This approach has significant implications for real-time crop disease detection as the use of lightweight models in combination with ViT structure can enable more efficient and accurate classification.

### 3.3 Challenges

Despite the progress in computer vision and artificial intelligence techniques for automated monitoring of crops for detection and identification of diseases, there are still some inadequacies. One of the major issues identified in the studies above is the difference in accuracy between the training and testing environments. Many existing studies have utilized identical datasets for both training and testing purposes, which inherently possess similarities and consequently yield high accuracy rates. For instance, a study conducted by Mohanty S.P (Mohanty et al., 2016). included a model that was trained using the Plant Village dataset, resulting in an impressive accuracy of 99.35%. However, when those model was tested against images sourced from online platforms, the accuracy dropped drastically to below 50%. This disparity highlights the need for a more comprehensive dataset. It is important to fix these problems to yield better accuracies from deep learning models. While existing methodologies have shown promising results with the pre-training data collected in lab controlled environments, the real-world scenarios pose a challenge due to variations in conditions. As a result, the models that are trained to a specific type of dataset tend to struggle when applied in real-world scenarios. To mitigate this issue, further construction of a dataset should be considered that contains diverse images ranging from lab-controlled environments to real-world scenarios. This would enable the development of more robust and adaptable models with improved accuracy across multiple environments. However, this is a demanding task and requires substantial resources, both in terms of time and effort.

An additional challenge is related to the stage and severity of the disease. According to Rumpf (Ioffe and Szegedy, 2015), the accuracy rate of disease identification fluctuates significantly, ranging between 65% and 95%. This variability primarily arises due to the nature of diseases, where they exhibit milder symptoms during the initial stages. As the disease progresses and its severity intensifies, the symptoms become more distinct, leading to higher accuracy in identification. The varying accuracy rates can be attributed to the fact that diseases in their early stages often present subtle or ambiguous symptoms, making them difficult to discern accurately. This poses a considerable challenge for automated monitoring systems that heavily rely on visual cues to identify and classify diseases. However, as the disease advances and its symptoms become more distinctive and prominent, resulting in improved accuracy in its identification. To address this challenge, researchers and developers need to focus on refining and training models to recognize the subtle signs and symptoms of diseases in their early stages. This would involve the collection and integration of datasets that encompass images representing different stages and severities of diseases. By incorporating a diverse range of samples into the training process, models can be trained to effectively identify diseases even when symptoms are not yet fully developed. By improving the recognition of subtle symptoms, automated monitoring systems can enhance their accuracy in disease identification throughout the entire spectrum of disease progression.

Another issue identified is the presence of diverse leaf shapes in plants that present a hurdle for image classification models (Xu et al., 2022) which results in discrepancies in their performance. One such crucial factor that influences the performance is the size of the images, which is closely linked to the distance between the camera and the plant at the time of capturing images. When the camera is positioned at a greater distance, the resulting images tend to be smaller in size. This aspect becomes particularly significant when it comes to disease recognition tasks, as smaller-sized images may not adequately reveal the diseased areas. As a result, the performance of the models suffers, leading to decreased accuracy in identifying and classifying diseases. To address these challenges, it is essential to develop image classification models that are robust and adaptable to variations in leaf shapes. Another factor is the variability in leaf shapes among different plant species that poses a challenge for image classification models. Each plant species exhibits unique leaf characteristics, such as variations in size, contours, and textures, making it more difficult for models to accurately classify them. As a result, the performance of these models may vary when confronted with plants that possess dissimilar leaf shapes. This can be achieved by incorporating diverse training datasets that encompass a wide range of plant species with varying leaf shapes and disease stages. Furthermore, efforts should be made to capture images at optimal distances to ensure that the resulting images are of sufficient size to accurately capture and identify diseased areas.

## 4 Open research problems and future directions

In current literature, it has been observed that although most of the existing CNN models are performing well under controlled settings, they are failing to produce satisfactory results in real-time scenarios. To obtain feature vectors from data, it requires systematic engineering and design expertise to recognize complex patterns in input data for subsystems.

The utilization of transformers for vision and image processing is promising but the development in this field is still in its nascent stages. Future research should prioritize exploring the potential of transformers and transfer learning as they have demonstrated encouraging outcomes for specific tasks compared to well-established CNN models. This is due to the extensive development that CNN models have undergone over the past decade, which has largely resolved their optimization challenges.

Future research should focus on creating better disease picture databases that include photographs of actual harvested crops in the field. Moreover, the increasing use of intelligent mobile devices highlights the need for lightweight model designs in potential research endeavors. Various studies, such as MobileNet and EfficientNet, are conducted to address this issue, and these versions are ideal for satisfying the needs of smartphone users due to their lightweight nature.

## 5 Conclusions

Deep learning methods have demonstrated significant potential in precision agriculture and automated disease detection. Our survey extensively reviewed several prominent databases specifically designed for deep learning in plant pathology, analyzing their outcomes and limitations. Furthermore, we thoroughly discussed both conventional approaches and cutting-edge technologies employed in plant disease detection. By examining the limitations of deep learning models, we uncovered an intriguing trend—these models experience a decrease in accuracy when transferred between different environments. Based on our analysis, we firmly conclude that there is a critical need for a concise dataset to improve the performance and accuracy of deep learning algorithms.

In addition to addressing dataset-related challenges, it is worth emphasizing the significance of incorporating transformer-based models in future research endeavors. As per our survey findings, the use of transformers has consistently yielded impressive results due to their capacity to capture intricate details. Moreover, their ability for few-shot learning offers a potential solution to mitigate the issues associated with acquiring large datasets that are accurately annotated. Therefore, we highly recommend that future research directions prioritize the exploration and integration of transformer-based models to further enhance the field of plant disease detection. Additionally, we advocate for the integration of transformer-based models as a means to address the challenges associated with gathering large datasets with accurate annotations. By adopting these recommendations, researchers can drive the field forward, leading to more efficient and reliable solutions for plant disease detection in real-world scenarios.

## Author contributions

ZS organized the data and literature and performed the statistical analysis. ZS also wrote the first draft of the manuscript and completed the final draft under the guidelines of all co-authors. AM contributed to the conception and design of the study and identified the literature sources as well as frameworks to be used for statistical analysis. JP directed the research, helped in the review process, and revised the manuscript multiple times. DH co-directed the research, provided input on the scope and limitations of the review, and corrected the final versions of the manuscript. All authors contributed to the article and approved the submitted version.
